# Chronic inflammation in polycystic ovary syndrome: A case–control study using multiple markers

**DOI:** 10.18502/ijrm.v19i4.9057

**Published:** 2021-04-22

**Authors:** Soumik Goswami, Subhadip Choudhuri, Basudev Bhattacharya, Rana Bhattacharjee, Ajitesh Roy, Satinath Mukhopadhyay, Sujoy Ghosh, Subhankar Chowdhury

**Affiliations:** ^1^Department of Endocrinology and Metabolism, Institute of Post Graduate Medical Education & Research and Seth Sukhlal Karnani Memorial Hospital, Kolkata, West Bengal, India.; ^2^Department of Biochemistry, Institute of Postgraduate Medical Education & Research and Seth Sukhlal Karnani Memorial Hospital, Kolkata, West Bengal, India.

**Keywords:** Polycystic ovary syndrome, Inflammation, C-reactive protein, Interleukin-6, Tumor necrosis factor, Microparticles.

## Abstract

**Background:**

Polycystic ovary syndrome (PCOS) is associated with insulin resistance and elevated risk of cardiovascular disease and diabetes. Chronic inflammation has been observed in PCOS in several studies but there is also opposing evidence and a dearth of research in Indians.

**Objective:**

To estimate chronic inflammation in PCOS and find its relationship with appropriate anthropometric and biochemical parameters.

**Materials and Methods:**

Chronic inflammation was assessed in 30 women with PCOS (Group A) and 30 healthy controls (Group B) with highly sensitive C-reactive protein (hsCRP), interleukin-6 (IL-6), tumour necrosis factor alpha (TNFα), and platelet microparticles (PMP). In group A, the relationship of chronic inflammation with insulin resistance, waist hip ratio (WHR) serum testosterone, and serum glutamate pyruvate transaminase (SGPT) were examined.

**Results:**

In group A, the hsCRP, TNFα, and PMP were significantly elevated compared to group B. However, IL-6 level was similar between the groups. In group A, PMP showed a significant positive correlation with waist-hip ratio and serum testosterone. IL-6 showed a significant positive correlation with insulin sensitivity and significant negative correlation with insulin resistance and serum glutamate pyruvate transaminase.

**Conclusion:**

PCOS is associated with chronic inflammation and PMP correlates positively with central adiposity and biochemical hyperandrogenism in women with PCOS.

## 1. Introduction

Polycystic ovary syndrome (PCOS) is the most common endocrinopathy in women of reproductive age (1). Women with PCOS are often insulin resistant leading to impaired glucose tolerance (IGT), metabolic syndrome, and dyslipidemia which markedly increases the risk of developing diabetes and cardiovascular disease (CVD) (2-5). Present body of epidemiological evidence, though inconclusive, suggests increased CVD-related mortality and morbidity in PCOS which is largely mediated through raised total and abdominal adiposity, with a possible interaction with PCOS-related hyperandrogenism (6). In several studies using markers like highly sensitive C-reactive protein (hsCRP), interleukin-6 (IL-6) and tumour necrosis factor alpha (TNFα), a link has been shown between chronic inflammation and development of cardiovascular disease and diabetes (7-9). Platelet microparticle (PMP) is a novel marker which has been shown to have a role in chronic inflammation, vascular dysfunction, and CVD (10). Several studies have evidenced the presence of chronic inflammation in PCOS but there are also contradicting studies (11-16). Nevertheless, it still remains unclear whether the primary factor affecting inflammation is insulin resistance, central adiposity, or androgen excess, although most of the evidence points to the first two factors (16, 17). Moreover, there are several studies on PCOS in Caucasians, but there is a scarcity of published data regarding inflammation in PCOS among Indian women with PCOS (18). Globally, there is meagre published data regarding PMP in PCOS (19, 20) but none from Asia in this regard.

Therefore, this study was designed with the primary objective of examining the existence of chronic inflammation in PCOS using PMP, hsCRP, TNFα, and Il-6 by comparing them with healthy control. The secondary objective of this study was to evaluate the correlation of inflammatory markers with obesity, insulin resistance, and testosterone in women with PCOS. This will help determine the presence and etiology of the risk for CVD and diabetes in Indian women with PCOS and identify targets for therapy and treatment monitoring.

## 2. Materials and Methods

### Participants

For the purpose of this case-control study, 30 women with PCOS (Group A) were selected from those attending the Endocrinology OPD of Institute of Postgraduate Medical Education & Research and Seth Sukhlal Karnani Memorial Hospital; Hospital and 30 controls (Group B) were selected from the hospital staff between January 2012 to December 2012. The inclusion criteria were: (i) women with PCOS diagnosed through the ESHRE/ASRM criteria, aged 15-40 yr after the exclusion of related disorders (serum prolactin, thyroid-stimulating hormone were done in all cases and 17 hydroxyprogesterone when indicated); (ii) Controls had regular menstrual periods with no clinical or biochemical hyperandrogenism. The exclusion criteria were: (i) Regular or recent medication intake; (ii) CVD or diabetes; and (iii) Recent surgery or trauma, infections, chronic systemic illness, addiction.

### Study design

Anthropometry was done to determine the patients' the women body mass index (BMI) and waist-hip ratio (WHR). Blood was drawn within 10 days of the onset of menses in oligomenorrheic PCOS women and controls and randomly in amenorrhoeic PCOS women after overnight fast to determine the fasting plasma glucose, fasting serum insulin, serum testosterone, PMP, hsCRP, IL-6, TNFα, and serum glutamate pyruvate transaminase (SGPT). Homeostatic model assessment of insulin resistance (HOMA-IR) was determined with the equation fasting glucose (mg/dl) × fasting insulin (μU/mL)/405 and quantitative insulin sensitivity check index (QUICKI) was calculated using the equation 1/[log (I (0)) + log (G (0))] {where fasting insulin is (I (0)) and glucose is (G (0))} with a calculator available at https://sasl.unibas.ch/11calculators-QUICKI.

### Measurement of PMPs

Platelets were obtained from venous blood, centrifuged at 350 g for 15 min with a buffer containing 145 mM NaCl, 2.7 mM KCl, 10 mM HEPES, 0.1% BSA, pH 6.5 (buffer A), followed by resuspension in the same buffer with pH 7.4 (buffer B). Platelet concentration was adjusted by buffer B to 2.0-3.0 X 105/μl in order to prepare PMPs. Platelets activation was done by calcium ionophore A23187 (10 μM) for 15 min at 37°C (14) and then centrifuged at 350 g for 15 min to allow sedimentation. Supernatant collection was done and centrifuged additionally at 16,000 g for 30 min. Washing of sedimented microparticles were performed once followed by resuspension in buffer B. Aliquots of 5 μl of each suspension were added to 40 μl of the same buffer containing additional CaCl${}_{2}$ (2.5 mM). Moreover, 5 μl anti-CD41-PerCP and anti-CD62P-PE were added to this mixture followed by incubation for 20 min at room temperature in the dark. Sample dilution with 300 μl of the same buffer was used to stop the reactions and ere immediately analyzed by flow cytometry. Acquisition was gated to include only the particles distinctly positive for anti-CD41-PerCP in order to differentiate platelets and PMPs from background light scatter.

### Measurement of other inflammatory markers

hsCRP present in the sample was measured by typical two-step sandwich enzyme-linked immunosorbent assay (ELISA) using the kit of Immuno Biological Laboratories (catalog no. IB59126, Minneapolis, USA), while IL6 and TNFα were measured by sandwich ELISA using the kit of Peprotech (catalog no. 900-K16, Rockhill, USA) according to the manufacturer's instructions.

### Ethical considerations

Appropriate permission from the Institutional Ethics Committee of IPGME & R and SSKM Hospitals, Kolkata was obtained before beginning the study (code: IEC 1099A). This study was non-interventional and informed consent was obtained from all cases and controls before inclusion.

### Statistical analysis

PMP, hsCRP, IL-6, and TNFα were compared between the study and control groups using unpaired *t* test with correction for BMI and WHR using multivariate analysis of covariance for confounding variables. The relationship of inflammatory markers with anthropometric and biochemical parameters were determined by bivariate correlation and Pearson's correlation coefficient was estimated. Data were analyzed using the SPSS Statistics for Windows, version 16.0 (SPSS Inc., Chicago, Ill., USA). A p-value of < 0.05 was considered as statistically significant.

## 3. Results 

Table I shows the age and anthropometric indices of the study population. The age of Group B was significantly greater than the cases, while both BMI and WHR were significantly greater in the group A and therefore the multivariate analysis of covariance was performed to correct these confounders. PMP, hsCRP, and TNFα were significantly greater in the cases compared to controls while there was no difference in the IL-6 between the groups (Table II). In group A, bivariate correlation was done to determine the Pearson's correlation coefficient (r) between inflammatory markers and biochemical and anthropometric parameters (Table III). A significant positive correlation was seen between the PMPs, WHR and serum testosterone. IL-6 showed a significant positive correlation with QUICKI and significant negative correlation with SGPT and HOMA-IR. There was no significant correlation of PMPs with BMI, SGPT, HOMA-IR, or QUICKI. Also, no significant correlation was observed between IL-6 and BMI, WHR, or serum testosterone. hsCRP and TNFα did not have significant correlation with any of the anthropometric or biochemical parameters.

**Table 1:** Age and anthropometric indices of study population


**Parameter**	**Group A (n = 30)**	**Group B (n = 30)**	**P-value**
**Age (yr)**	22.20 ± 8.32	27.60 ± 4.51	0.003
**BMI (kg/m ${}^{2}$)**	27.48 ± 4.41	24.18 ± 2.08	0.001
**WHR**	0.87 ± 0.06	0.82 ± 0.04	≤ 0.001
BMI: Body mass index, WHR: Waist–hip ratio, Student's *t* test was used to compare the age and anthropometric indices between groups A and B			

**Table 2:** Inflammatory markers in the study population


**Parameter**	**Group A (n = 30)**	**Group B (n = 30)**	**P-value**
**PMP/μL**	204.4 ± 86.27	115.5 ± 37.24	0.000
**hsCRP (mg/L)**	0.54 ± 0.32	0.31 ± 0.23	0.012
**IL-6 (pg/ml)**	7.15 ± 4.09	7.7 1± 2.92	0.117
**TNF α (pg/ml)**	21.56 ± 11.15	16.73 ± 6.50	≤ 0.001
PMP: Platelet microparticle, hsCRP: Highly sensitive C-reactive protein, IL-6: Interleukin 6, TNFα: Tumor necrosis factor alpha Multivariate analysis of covariance was used to correct the varying baseline parameters (age, BMI, WHR) to avoid confounding and thereafter compare inflammatory markers between groups A and B			

**Table 3:** Correlation between inflammatory markers and biochemical and anthropometric parameters in group A


**Parameter**	**PMP**	**hsCRP**	**IL-6**	**TNF α**
	r	–0.041	–0.100	0.309	–0.143
**BMI**	p	0.830	0.599	0.097	0.451
		r	0.402	–0.244	–0.224	–0.152
**WHR**	p	0.027	0.194	0.235	0.426
		r	–0.136	–0.229	–0.617	–0.168
**SGPT**	P	0.474	0.223	0.000	0.375
		r	0.249	–0.214	–0.432	0.115
**HOMA-IR**	p	0.185	0.257	0.017	0.547
		r	–0.119	–0.039	0.615	0.174
**QUICKI**	p	0.531	0.836	0.000	0.359
		r	0.660	–0.286	–0.080	0.199
**TES**	p	0.000	0.125	0.673	0.293
	BMI: Body mass index, WHR: Waist–hip ratio, SGPT: Serum glutamate pyruvate transaminase, HOMA-IR: Homeostatic model assessment of insulin resistance, QUICKI: Quantitative insulin sensitivity check index, TES: Serum testosterone, PMP: Platelet microparticle, hsCRP: Highly sensitive C-reactive protein, IL-6: Interleukin 6, TNFα: Tumor necrosis factor alpha, Bivariate correlation was done to determine Pearson's correlation coefficient (r) between obesity parameters, insulin resistance markers, and serum testosterone with markers of chronic inflammation in group A					

## 4. Discussion

In the present study, we evaluated 30 women with PCOS and 30 controls. The cases were included based on the Rotterdam criteria, while the controls were women with regular menses and no clinical or biochemical evidence of hyperandrogenism. PMP, hsCRP, and TNFα were significantly higher in the cases compared to the controls, while there was no significant difference in the IL-6 levels between the two groups. These findings corroborated with those of several other studies in published literature.

Similar to our study which showed elevated PMP in women with PCOS compared to controls, two separate studies conducted in Greece showed that PMP were elevated compared to the BMI and WHR ratio-matched controls in both lean and obese women with PCOS (19, 20).

Kelly *et al* first demonstrated increased CRP levels in PCOS compared to controls in 2001 and several subsequent studies have shown similar findings (12). In a Korean study, it was showed that in lean women with PCOS, the hs-CRP level was higher in the impaired glucose regulation group than in the normal glucose tolerance group (21). Another study showed increased CRP levels in women with PCOS compared to controls (22). In a study from India, hsCRP was elevated in women with both newly diagnosed and on-treatment women with PCOS compared to healthy volunteers (23). Circulating CRP was shown to be 96% higher in women with PCOS compared to controls in a meta-analysis of 31 articles. This finding held good even after excluding 5 studies with mismatched body mass and/or obesity frequency between women with PCOS and controls (11). However, in another Indian study, the hsCRP level was not significantly higher in women with PCOS compared to controls (18). Overall, as has been discussed, the majority of studies in published literature refer to increased levels of hsCRP in PCOS which was what was found in our study.

Serum TNFα was found to be significantly higher in women with PCOS compared to controls in two separate studies from India and Turkey (13, 22). In an American study, TNF-α was significantly increased in obese adolescent girls with PCOS (BMI ≥ 27 kg/m${}^{2}$) when compared with BMI and age-matched controls (24). These findings are in agreement with our study which similarly showed an increase in TNFα in women with PCOS. However, a meta-analysis of nine studies showed no statistically significant difference in TNF-α levels between PCOS and controls (25).

A meta-analysis of 10 studies revealed no statistically significant difference in IL-6 between PCOS and controls (25). These findings are similar to our study where no significant difference was noted in the IL-6 level between PCOS women and controls. However, there are published studies with dissimilar findings. In an Indian study, women with both newly diagnosed and on-treatment PCOS exhibited higher plasma concentrations of IL-6 than healthy volunteers (23). In an Italian study, IL-6 was found to be significantly increased in all insulin-resistant PCOS, independent of BMI, but there was no increase in serum TNFα levels (26). Likewise, a study from Taiwan showed significantly elevated levels of IL-6 in PCOS women compared to controls (27).

In our study, there was a significant positive correlation between PMPs and WHR and serum testosterone. In a study in lean women with PCOS, elevated plasma PMPs in PCOS women correlated with serum testosterone levels suggesting that hyperandrogenemia is primarily responsible for the elevated plasma PMPs in women with PCOS. However, it did not correlate with adiposity or impaired glucose metabolism in this study (19). In another study in obese women with PCOS, plasma PMPs did not correlate with the markers of insulin resistance and serum testosterone. However, they were higher in the group with more insulin resistance, higher serum testosterone, and an increased number of follicles suggesting an association with androgens and insulin resistance (20).

In our study, neither hsCRP nor TNFα had any significant correlation with any of the anthropometric or biochemical parameters measured. This finding was similar to a study which reported no correlation between TNFα and BMI in adolescent girls with PCOS (24). In another study from Brazil, hsCRP was higher in obese women with PCOS compared to lean women with PCOS but TNFα was similar between the two groups (16). However, a study from Turkey showed the presence of significant correlation of both CRP and TNFα with free androgen index (22).

In our study, IL-6 showed a significant positive correlation with QUICKI and a significant negative correlation with SGPT and HOMA-IR. An animal study comparing IL-6-/- mice with littermate control mice demonstrated that mice in the absence of IL-6 develop glucose intolerance and insulin resistance. Also, IL-6-/- mice showed signs of hepatic inflammation (28). The role of IL-6 in T2DM development is still an unanswered question. Although studies point to the role of IL-6 in impairment of insulin signaling in adipocytes in vitro, a similar finding has not been demonstrated in vivo. Skeletal muscle insulin resistance has been shown to be unrelated to long term IL-6 stimulation and further studies are necessary in this area (29). In our study, IL-6 did not have a significant correlation with obesity parameters but in another study, IL-6 was significantly greater in obese women with PCOS compared to lean women with PCOS (16).

The discrepancies noted between our study and certain studies in published literature could be due to differences in inclusion criteria, ethnicity, and sample size, and this controversy may be resolved in the future with studies including a larger number of women with PCOS.

## 5. Conclusion

We conclude that PCOS is associated with chronic inflammation and that PMPs correlate positively with central adiposity and biochemical hyperandrogenism in women with PCOS. Targeting central adiposity and hyperandrogenism with our present therapeutic armamentarium could be a possible avenue for reducing cardiovascular risk in women with PCOS with prospective randomized controlled trials necessary to provide the accurate answer in the future. Also, PMPs could serve as a novel futuristic therapeutic target to minimize inflammation and CVD in women with PCOS.

##  Conflict of Interest

There is no conflict of interest.
